# Inner Peace: Evaluating a Complementary Program Promoting Intra-Personal Peace at Adelaide Women’s Prison, Australia

**DOI:** 10.1177/0306624X241246099

**Published:** 2024-04-24

**Authors:** Anne Turner, Natalie Thomas, Helena Menih, Anthony Collins

**Affiliations:** 1 La Trobe University, Bundoora, VIC, Australia; 2 The University of Queensland, Brisbane, Australia

**Keywords:** complementary program, inner peace, prison, women, rehabilitation

## Abstract

The Peace Education Program, created in 2012, is a complementary program with potential to supplement official rehabilitation interventions offered in correctional centers. The program promotes “inner peace” as an innate and universal human resource, but whilst inner peace is a key concept in positive psychology and the Good Lives Model, there is a paucity of research regarding how to operationalize and evaluate this concept. The program had not previously been the subject of independent theoretically-informed research. Drawing on a mixed methods study conducted in Adelaide Women’s Prison, this article explores the impact of the program on participants’ learning regarding inner peace. Participants reported a greater understanding about inner peace, which they described as contributing to a stronger sense of their identity, enhanced self-esteem and increased self-regulation skills, resulting in reductions in impulsivity and reactive aggression. The quantitative data indicated there was a significant increase in participants' subjective ratings of inner peace before the program (*M* = 12.08) and post-program completion (*M* = 14.00) (*p* < .001). Growth in affect-regulation and anger-management skills may contribute to reductions in offending.

## Introduction

The landscape of correctional rehabilitation is evolving as societies increasingly recognize the multifaceted nature of offender rehabilitation. While official rehabilitation interventions in correctional centers play a crucial role in the reintegration process, there exists a growing acknowledgment of the need for supplementary programs that can enhance and diversify the impact of traditional approaches. The theoretical underpinning of mainstream programs delivered by corrective services utilizes the evidence-based rehabilitation paradigm, the Risk Needs Responsivity model (RNR) (Andrews & Bonta, 2010; Bonta & Stephen Wormith, 2013). However, the complex and varied needs of individuals within the criminal justice system often require a more collaborative, individualized and holistic approach beyond the framework provided in the RNR model. This has led to the emergence of strengths-based models of offender rehabilitation, such as the Good Lives Model (GLM) (Ward et al., 2012; Weekes et al., 2013), which seek to enhance offenders’ wellbeing. Positive approaches to offender rehabilitation also inform the wide range of complementary programs that have burgeoned in the carceral environment in recent decades, which seek to supplement and reinforce the efficacy of official rehabilitation interventions by offering participants means for personal development and positive mental health.

The focus of this paper is an exploratory study which investigated the impact of the relatively new complementary Peace Education Program (PEP) in Adelaide Women’s Prison (AWP) in Australia. The program was created in Dominguez State Jail in Texas and focuses on encouraging the development of internal psychological resources. The content of the program is largely based on the words of Prem Rawat.^1^ The program uses mixed media for the content of the 10 modules. The modules offer encouragement to participants to respect themselves, to identify their inner strengths, to develop the perception of themselves as agents of their own destiny, to acquire a more positive sense of their own identity, and to experience and take responsibility for inner peace (The Prem Rawat Foundation [TPRF], 2022). Participants are self-selected and voluntary.

Although there have been evaluations of the program with correctional populations (TPRF, 2022), these evaluations were commissioned by the administering foundation and lack a theoretically-informed methodological basis for understanding how the program might impact on desistance from offending. This study is the first independent, theoretically-informed, and comprehensive analysis of the program in the carceral environment, and also the first evaluation investigating the role of gender in the PEP to date. More specifically, the study analysed whether participants’ responses to the program resulted in any cognitive and behavioral change that might indicate the potential of the program to impact re-offending. This required a comprehensive review of the literature to develop instruments to measure the impact of the PEP on primary aspects of identity potentially related to offending (including agency, self-esteem and inner peace).

Mixed methods were used to assess the impact of the program on a sample of 15 incarcerated women. Quantitative surveys were administered at program commencement and program completion. Qualitative interviews were conducted after program completion, as well as with six facilitators. This paper presents the findings focused on the impact of the program on inner peace, a concept which has been largely under-explored in the literature, although it is recognized as a “primary good” within the Good Lives Model of offender rehabilitation.

## Literature Review

### Inner Peace

The definitions and dimensions of the construct of inner peace are the subject of debate in the literature by various authors. According to Fox (2013), peace as both a concept and a reality is little understood (or experienced). Fox emphasises that peace has been defined in terms of negatives, such as the absence of war and hostilities. He advocates creating more positive definitions of the attributes of peace. The debate as to whether peace is predominantly internal or external, Fox suggests, is a false dichotomy, in that they are normally inter-related.

Definitions of inner peace often draw on the concept of internal states or emotional regulation. Chu et al. (2014, p. 4) define inner peace as “freedom from emotional toil and stress,” which relates the concept of inner peace to more scientifically established psychological measures. Similarly, Serie et al. (2021) relate the primary good of inner peace to “emotional self- regulation and achieving a state of emotional balance” (p. 4), and to an attunement to the emotions of oneself and others. For the means of enhancing inner peace, they suggest exercise, meditation, counseling, or other activities that facilitate emotional regulation. Fortune et al. (2014) propose that the converse of inner peace is defined by emotional dysregulation, which Marshall and O’Brien (2013) explain as typically arising from maladaptive coping responses to life’s problems. They describe deficiency in coping skills, which they state have been identified as criminogenic, as appearing “to be a significant factor in producing emotional and, therefore, behavioural dysregulation” (Marshall & O’Brien, 2013, p. 287).

In the literature, inner peace is relatively undervalued and under-researched (Fox, 2013; Xi & Lee, 2021), perhaps because it has traditionally been associated with spiritual rather than scientific traditions. Sims et al. (2014) could find only 22 references to inner peace in a search of PsycINFO, and one reference to intrapersonal peace. They state that psychology has failed to embrace the study of intrapersonal peace, which is the gap their book seeks to start to address (p. 2). Fisher (2011) analyses the inter-connectedness of spirituality and well-being, offering a definition of spirituality as being “concerned with a person’s awareness of the existence and experience of inner feelings and beliefs, which give purpose, meaning and value to life” (p. 20). Fisher then identifies four components: internal (defined as the search for identity and self-worth), communal/inter-personal relationships, environmental, and transcendence. Sun (2013) also offers definitions of “mental peace” (p. 464) as essential for the maintenance of inner peace and the experience of joy, and the ability to heal “past emotional hurts and extricate oneself from fear, anxiety, and depression.”

Floody (2014) remarks on how a greater understanding of the factors relating to inner peace would offer the opportunity to “address the underlying causes of anxiety, depression, and dissatisfaction with life” (p. 107). Floody (2014) also suggests that a greater understanding of the factors relating to inner peace would offer the opportunity to “address the underlying causes of anxiety, depression, and dissatisfaction with life” (p. 107). His preference is to use the word “serenity,” which he operationalizes using the “Viterbo Serenity Inventory” (on the dimensions of: higher power, harmony, positivity and lifestyle).

The importance of inner peace in offender rehabilitation was elevated by being identified as a “primary good” in the Good Lives Model (GLM) (Ward et al., 2012; Weekes et al., 2013). Much of the research around programs implementing the GLM has however focused on sexual offenders (Barnett & Wood, 2008; Marshall et al., 2013; Marshall & O’Brien, 2013), restricting the generalizability of such studies in terms of the larger population of non-sexual offenders. In studies of sexual offenders there is often a clear correlation between the offending and dysfunctional attempts to obtain intimacy. Barnett and Wood (2008) assessed 42 untreated sex offenders around three operationalized components of the GLM which they saw as being particularly correlated with sexual offending: agency, relatedness, and inner peace. They found an inter-relationship between the self-reports of their participants and dysfunctional quests to fulfil those primary goods.

The “small-scale narrative enquiry” of Hamilton (2016), building on the work of Farrall et al (2014), sought to emphasise the importance of the role of emotion (including shame, an obstacle to making peace with oneself) in changing cognitions and thus attaining pro-social identities. The research subjects frequently identified the need to make “peace for what they had done—sometimes with God, sometimes with others, but always within themselves, of finding inner peace and peace of mind, happiness and inner contentment” (p. 37).

Attempts to operationalize and measure the concept of “inner peace” tend to focus on internal mental states. The World Health Organisation (WHO, 1998) Quality of Life subsection on Spiritual Religious and Personal Beliefs (WHOQOL SRPB) report offers the definition of “inner peace/serenity/harmony” as the extent to which people are at peace with themselves. The report describes inner peace as a “highly desirable condition” as it helps people cope when things go wrong. For its simplicity and user-friendliness in meeting the quantitative element of this study, the WHOQOL-SRPB four-item scale was selected in order to operationalize the concept of inner peace.

Xi and Lee (2021) also highlighted the lack of attention in the literature in conceptualizing and/or measuring inner peace, which they define as a “fundamentally balanced mental state” (p. 436). Xi and Lee (2021) developed a scale to measure inner peace around three dimensions they consider vital components: acceptance (which they relate to loss), transcendence (which they relate to ceasing a quest for pleasure or hedonism), and calmness.

Traditionally, peace education programs focus on social interdependence and teaching students skills promoting cooperation and consensus to enable conflict resolution (Johnson & Johnson, 2006, 2011). The Peace Education Program does not appear to seek to emulate more traditional peace education programs focusing on interpersonal interaction, and has far greater affinity with other complementary programs such as yoga or mindfulness, in that the PEP encourages the individual to focus on their inner resources.

### Complementary Programs

Complementary programs include art therapy, music therapy, and yoga and meditation programs, offering a means for personal development. Yoga/mindfulness/meditation programs lead the field in terms of specifically focusing on the well-being of the individual and in terms of the volume of research data on individual programs (Bartels et al., 2019; Danielly & Silverthorne, 2017; Hauzinger, 2018; Samuelson et al., 2007;) and meta-analyses (Auty et al., 2017; Wimberly & Xue, 2016; Muirhead & Fortune, 2016; Han, 2022). Whilst a direct link between mood, well-being and offending has yet to be proven by empirical research, the literature generally indicates improvements in mood (Bartels et al., 2019; Danielly & Silverthorne, 2017), and suggests improvements in motivation may facilitate greater openness to benefitting from the Risk Needs Responsivity (RNR)-based programs (Auty et al., 2017; Bartels et al., 2019; Muirhead & Fortune, 2016).

Focusing on the breath is a fundamental aspect in most practices of meditation, mindfulness and yoga (Unrau & McCormick, 2016). Whilst the Peace Education Program also encourages connecting with the breath as a means of accessing the peace within, the emphasis is wider-ranging in that it invites participants to consider their lives (and the human condition in general). This study extends the limited research on inner peace in the carceral environment to the relatively unresearched complementary Peace Education Program. It undertakes a more rigorous exploration of the experiences and impacts of the program and examines the participants’ understandings of how and why it may be effective. It focuses on women as a historically under-researched group who are subject to a significant range of intersectional disadvantages, which have a clear relevance to their carceral status (Brennan et al., 2012; Daly, 1994; Fader & Traylor, 2015; Latessa et al., 2020; Scott et al., 2019). The issue of the gender and cultural neutrality of the program was canvassed with participants, with no negative responses. Foremost, however, this study provides an analysis of participants’ words regarding their experience of the program relating to themes of inner peace and self-identity change that may be relevant to desisting from offending.

## Methods

The study investigated the impact of the Peace Education Program at Adelaide Women’s Prison (AWP), with one focus being specifically on the impact of the program on participants’ experience of inner peace. Mixed methods were used in order to obtain as comprehensive results as possible. Fifteen program participants and six facilitators were interviewed remotely by phone due to the stringent Covid-19 pandemic restrictions in place at the time. Participants were self-selected; everyone was dis-identified using pseudonyms.

Fifteen participants completed the survey measures which were selected to best assess the impact of the program. Whilst the quantitative part of the research included other aspects deemed to have potential relevance to desistance (such as agency and self-belief), for the purpose of this paper only the information relevant to the data collection regarding inner peace will be discussed. Additionally, even with a small sample, quantitative results are important to consider in combination with the qualitative results.

### Data Collection

Ethics approval was obtained from La Trobe University (HEC20462) and approval received from the South Australia Department for Correctional Services. Data was collected during 2021. Variations had to be sought in order to progress the research despite the restrictions due to Covid-19 at the time. These included switching to phone interviewing, and obtaining the assistance of the primary facilitator in presenting the participant information and consent forms (PICFs) and the survey measures. The quantitative data was collected in two stages: at pre-commencement (T1) which provided the baseline before participants commenced the program, and post-completion (T2), enabling assessment of whether participants reported any attitudinal change after completing the program. To ascertain the participants’ understanding of “peace” and operationalize this concept, the study used the WHOQOL-SRPB, a four-item assessment scale which relates specifically to inner peace (Tendhar, 2014). The WHOQOL-SRPB measure is open and for public use. It consists of eight facets or sub-scales, of which one is inner peace. Given that this sub-scale was not created as a stand-alone measure, it has not been therefore empirically validated as such.

With the restrictions consequent on the Covid-19 pandemic, the qualitative data collection was only logistically possible at T2. The importance of confidentiality was emphasised, which is particularly essential with vulnerable subjects who need to feel safe in disclosing information. Participants (including the facilitators interviewed) were advised that their names would be de-identified using pseudonyms selected by the researcher. Facilitators distributed and collected consent forms and forwarded these to the primary researcher.

Qualitative interviews were conducted with 15 participants of the program, and six facilitators of the program. The participants were informed that a recording device was being used in order to collect their words accurately, and that the interviews would then be transcribed, to which they gave consent. The data was orthographically transcribed almost immediately following the recording. The qualitative part of the research also included the thematic analysis of the program materials used by the facilitators at Adelaide Women’s Prison.

### Participants

Eleven women had completed the Peace Education Program Collection, where the 10 modules consist of videos of approximately 25 min duration, with two modules being presented per session. Four women had completed PEP 3, where the modules are effectively twice as long, and conducted over a 10-week period. Seven of the participants identified as Aboriginal (their choice of words), one participant identified as Maori, and one identified as Persian. The remaining six participants identified their ethnicity as Australian.

Six facilitators of the program were also interviewed; two were female and four were male. They were mostly retirees from professional backgrounds. The team leader (Bill) was a former GP whose appreciation of the need for independent research was integral to his high level of cooperation in progressing the study despite Covid-related disruptions.

### Analysis

#### Qualitative Data

Analysis for this project was conducted by the first author; this article reports only on the results that were relevant to inner peace from the larger project of the Peace Education Program evaluation conducted by the first author. The content of the interviews of the 15 participants and the six facilitators was transcribed and analysed by the first author using NVivo. The themes (which also included agency, self-esteem, belief in redeemability, generativity and gratitude) had been to some extent pre-identified from the literature as potentially relevant to self-identity change that could be conducive to desistance from offending. Nevertheless, the coding evolved in a semi-organic way, and thus utilized both inductive and deductive coding. This data analysis followed the recommendations of Braun & Clarke (2006) with the stages of thematic analysis they identified (p. 87). They describe the identification of themes as “. . .where the interpretative analysis of the data occurs” (p. 88). Following their distinction, this analysis was both data and theory-driven. As the coding progressed, the sub-codes became clearer in order to encompass all the raw data, and thus under the over-arching themes, additional sub-themes were identified as they arose.

A deductive thematic analysis was also conducted of the program materials in use at Adelaide Women’s Prison, specifically PEP 3 and PEP Collection (PEP CL) and any additional materials available to participants. The thematic analysis of the program material was handled differently from the thematic analysis of the participant interviews because of the inherent difference in the data sets. The material in the program media is relatively static in nature, having been pre-recorded. With the materials, in order to address the main research question the themes were pre-identified following the information culled from the literature review regarding aspects of identity potentially relevant to desistance from offending.

#### Quantitative Analysis

All quantitative data from the surveys completed by the participants was analysed using the Statistical Package for Social Sciences (SPSS) software version 29.0. Paired samples *T*-tests were used to determine if there was a change between the pre- and post-program scores on the WHOQOL- SRPB, with a *p*-value of < .05 considered significant. Internal reliability of the scale was tested using Cronbach’s alpha, demonstrating excellent internal consistency (Cronbach’s α = .95).

## Results

This article reports only on the aspects of relevance to inner peace, with the results of the survey items that measured subjective feelings of inner peace being discussed. Similarly, the qualitative analysis both of the program content and of the interviews focuses selectively on the issue of inner peace, the subject of this paper.

### Quantitative Results

The null hypotheses stated that there would be no significant difference in inner peace as measured by the WHOQOL between T1 and T2. A paired samples *t*-test indicated that subjective feelings of inner peace improved significantly between the pre-measure (*M* = 12.08, *SD* = 4.68) and the post-measure (*M* = 14.00, *SD* = 3.95) (*p* < .001).

### Qualitative Results

Quantitative analysis of the survey measures indicated that subjective feelings of inner peace improved significantly between the pre-and post-measure. The qualitative analysis complemented the quantitative analysis to investigate how the program impacted on participants’ feelings of inner peace.

#### Program Materials

The analysis conducted of the materials used in the Peace Education Program at Adelaide Women’s Prison consisted primarily of the PEP 3 materials (10 modules over 10 weeks) and PEP CL (10 condensed modules over 5 weeks). This also included the film “Inside Peace” (shot in 2014 Dominguez State Jail, TX, USA.) which is used at AWP as an introduction to the program, and the books “Hear Yourself” (Rawat, 2021) and “Splitting the Arrow” (Rawat, 2016). Most participants in this study attended the PEP CL, which appeared more user-friendly for participants with shorter attention spans, and better adapted to the high level of attrition due to the transience of the population.

The program materials draw on public talks by Prem Rawat, as well as: interviews with him by media personalities; interviews with participants in the program; interviews of the public, and interactions between Rawat and specific audiences. The tone is supportive and encouraging rather than critical, with the use of humor also serving to make the content non-confrontational. Animated stories and music with other visual effects are used to enhance the verbal content. The format for each module is the same: short videos about the module topic; time for reflection on the content is included during the sessions, with the option for facilitated discussion. A workbook and/or handout which briefly lists the contents of each module, plus supplementary material, is also given to participants, and includes a space for comments about whatever they have understood. Stories sometimes feature in the material either in cartoon form in the videos or in text form in the supplementary material. Participants are invited to complete a simple feedback form on completion of the program.

The clear message of the program is that peace is an innate, internal resource, available to everyone, and that peace is the responsibility of each individual who recognizes its value. Whilst the first module is entitled “Peace,” however comments about inner peace occur throughout the material. In Module 1 peace is described as an unchanging, fundamental need inside all human beings intrinsic to well-being, stressing that what we are looking for is already inside of us and thus accessible to everyone should they choose to experience it. This module explores the concept of peace including interviews with various members of the public, canvassing their understandings of peace—which are predictably varied. Connecting with the breath is advocated as a means of accessing the peace within.

In PEP CL (Module 1) Rawat states that “peace never has been and never will be far from you—(it) lies in the heart of every single human being.” He emphasises that creating a world at peace not only starts with the individual, but is also the responsibility of each individual. This is echoed in PEP 3 (Module 1), where Rawat describes it as being “incumbent” to use our individual resources to create a world at peace.

In the movie “Inside Peace” (Fitzpatrick & Fitzpatrick, 2014) one inmate expresses “you know, we’re all here seeking the end to the pain that has characterized our lives. To see everybody at peace in prison—would shock the world” and suggests that if it is possible to find peace in prison then surely it is possible to achieve peace anywhere. In the book “Hear Yourself,” Rawat (2021) references inner peace as not being “dependent on or defined by other people, or by anything outside you” (p. 16). Rawat (2021, pp. 184–185) parallels the experience of living without internal peace to incarceration:

Being separated from inner peace is a terrible life sentence, whether inside or outside prison. Fears, expectations, and prejudices: they become like walls, doors, and bars. And the person who is making your life miserable within that prison is you.

#### Interviews with Participants and Facilitators

This section firstly describes the more general comments made by participants regarding inner peace. It is followed by specific aspects of behavioral change relevant to this concept that participants identified from the program, which are potentially relevant to reductions in offending. Given that the program is voluntary, and participants are self-selected, this may mean that there is some bias in participants’ readiness for change.

Some participants described the focus of the program on “peace” and self-improvement as a motivator for attending the program. One of the participants, Annette, explained: “I just heard the words “inner peace,” and I was there. I figured no bad can come ever from having inner peace, and I’ve been through a lot of traumatic experiences in life over the years so yeah I was definitely keen.” This assumption of bias is countered to some extent by participants who had “signed up” simply because they wanted to get “as many courses under my belt as possible” (Jess), or who hoped that the program would prove to be less boring than other options available.

When discussing her hopes for the positive effects of inner peace, Martha expressed: “It would make me a better mum, it would make me a better daughter, it would make me a better partner.” India explained that “it would be a great, great pleasure to feel peace in my life” and further commented that it would deepen her conviction that she would never return to prison. Layla’s take on experiencing inner peace was that it could allow her to make positive life choices since she would have an enhanced sense of her own values and dignity. As she explained:

If I have the inner peace, of course I wouldn’t want to get revenge of people like that hurt me before, or go and do things that is against my values and against my dignity, so I would let it go, I would say “don’t worry about it, I am staying on this path,” and I will choose another way, the peaceful life with the peace.

In Olivia’s opinion, acquiring inner peace would help her “relax, to calm down, to be, you know, at ease” within herself, and that this would have a knock-on effect in her dealings with others, becoming “a better person to everybody else,” but also becoming more confident and assertive in her communication.

Several participants described that they felt more peaceful or “calm” after doing the program. Some even spoke of experiencing a collective sense of peace which occurred during the sessions which they felt they needed, perhaps particularly in their current situation. Most, however, spoke of how this core concept of the program had helped change their attitudes and behaviors. Jess commented that she acquired a deeper understanding of what the words about having “inner peace” actually meant and how this could relate to her life.

Naomi explained how she was already attempting to action inner peace whilst incarcerated, and how for her this was related to forgiveness. Clair was able to look beyond individual experience to hypothesize that experiencing inner peace could result in external peace and, ultimately, a world in peace “because if people had inner peace they would become happy with each other and in themselves, and there’s so much less conflict when it comes to that.” She was also able to identify how the program had already helped her to come to terms with her previous behavior, and to deal with her previous experiences of trauma.

Dana stated that the program had taught her to learn to love herself and to change the way she thought about others, to learn to want to “give back,” and she related this to having acquired a sense of inner peace within herself. Layla described how reflecting on the content of the modules enabled her “to reach to a point to find the peace inside me, a very improvement in my life, yes.” She also linked finding inner peace to acquiring more of an appreciation for her life, and to the realization that offending was incompatible with inner peace. Harriet reflected on her past when thinking about inner peace: “It just taught me ways to forgive myself and to be at peace with what I’ve done and be at peace with the fact that I am in jail.”

#### Behavioral Change

Whether learnings from undertaking the Peace Education Program result in any long-term behavioral change is the key issue regarding the potential of the program to impact re-offending. Most participants spoke of some modified responses in their behaviors that they had already noted in response to the program. These can be broken down into three categories of behavioral change relating to inner peace that they described: reductions in anger and inter-personal conflict; reductions in impulsivity; and feeling more secure in themselves and less reliant on the opinions of others.

These categories are not mutually exclusive, with an increase in affect control being linked with expressions of greater objectivity and self-regulation, acquiring the ability to take a more detached perspective and not impulsively responding with anger and aggression. Most participants described becoming less reactive. For example, India described feeling calmer: “I’m not so highly strung all the time, I don’t stress as much now about things.”

Regarding anger, Grace stated: “I get on more better with people, yep, than before the program. I’m not violent anymore. I don’t get angry with other females like I did before, despite a lot that I don’t know.” Kym linked anger to the experiences of Aboriginal people, describing how many people in her community experienced anger and frustration at their treatment, and she believed that the program could help her lead them to a greater sense of peace. Facilitator Bill provided an insight into a participant who acknowledged her anger was the issue, and that she realized that she needed to change. His recollection of her words was: “it’s my anger that keeps bringing me back to jail. It’s MY anger, I know why I’m angry, it’s not helping me or anybody else, it’s wrecking my life, I’m going to do something about it.”

The examples of reductions in inter-personal conflicts described by participants were relatively trivial verbal exchanges rather than involving any physical violence, nevertheless some participants realized that these conflicts were a threat to their “inner peace.” Clair described one such incident:

I live with someone that’s quite young and thinks they rule the world. And we had an argument about something the other day, and I thought “wow, this is just stupid!” and I just walked away and thought, “I don’t even care, there’s no point in arguing over nothing,” and I wasn’t probably thinking about the course when I said it but now that I look back on it, I think I wouldn’t have done that before.

Other participants also described how the program helped them manage interpersonal conflicts. Layla also reflected on her experience:

There was an argument with another prisoner, and I just stopped anger, and I said “no, this is not me,” I just let go, and forget about it. . .and I made peace first within myself and then to them, I made peace with myself and stopped myself being angry, and then made peace between us. . ., we finished as friends.

Annette described a more general situation at her place of work—the prison kitchen—where relationships could become strained: “it can be a bit bitchy sometimes over there, so it’s taught me that those conversations and that are not important to me, I can just keep on track with what I’m doing, and not worry about all that.” She described how in general she had managed to become more accepting of situations and more philosophical about them, such as her parole hearing coming up in two days. She also expressed that the program “taught me how to not get so worked up and emotional about things that I don’t need to.”

The scenarios described above by participants involved them in “stepping back” in order to re-appraise a situation where they might previously have responded more impulsively with aggression. Dana described that the program had changed “the way I respond to conflict.” Bernie expressed how she wanted to stop “. . . dwelling on things, and being angry about things,” and to use the learnings she had made in the program to get her sons “to reflect on their choices instead of jumping to conclusions.” Similarly, Martha described a situation which could previously have led to escalation or continued resentment, where she was able to adopt a more detached perspective “instead of me erupting and losing my shit like I probably would.”

Jess related that acquiring inner peace would enable her to make less impulsive life choices, acknowledging that she had previously experienced “a lot of reckless behaviour.” For Faye, stopping and focusing on the breath would she believed make her less likely to act impulsively, as she phrased it: “not letting anger get to you, like try and breathe and just think about something before you get into trouble.”

Several participants expressed that achieving a greater sense of security in themselves would help them be less dependent on the opinions of others. For Grace, experiencing inner peace: “would help me heaps, cos I need to find peace in myself, you know, I’m typically worrying about other people, what they think and say about me, I’m not going to worry about all that.” Grace also expressed the insight that she had realized that she needed to be at peace with herself in order to be at peace with others.

Clair described how the program had given her a stronger sense of her own identity “after watching and listening to Rawat, it’s a different process of being on your own now, like I’m more content, not with what I’ve done, but with who I am.” She related inner peace to being more secure in her self-identity: “You’ve got routines in here and out there, but when you sum it all up, you really can’t function unless you actually have peace, that inner strength, and guidance within your own sense of where you know you’re going.”

Harriet described being reminded that she did not need the company of others to avoid boredom, she could be content in her own company “and not having to involve in different groups of people just to feel accepted.” In a similar vein, India described how previously “I always just had to have people around me to make me feel good, but now I can sit by myself and feel good.”

Layla in response to a comment from the interviewer suggesting that she had already decided before the program that she did not want to come back to prison:

100%, but this is more—it makes me reach to the inner peace that why would I do these, why as a human that I have lots of respect and value for myself, I have dignity for myself, so why would I go and commit a crime that is not me, you know?

As described in the literature review, inner peace has held a marginal status in relation to desistance from offending (Xi & Lee, 2021), despite the Good Lives Model according it the status of one of the seven primary goods. Layla’s comment, however, would indicate to the contrary its fundamental significance. She describes how acquiring some experience and understanding of the value of inner peace can affect decisions and behavior—including offending behavior. Her words also indicate that there is some connection between inner peace, self-identity, and self-respect/self-worth—with which offending is not consistent or congruent.

In relation to the experience of incarceration, Harriet recommended that it continues to be used in prisons as a means of helping “prisoners become at peace with the fact they are in jail. I do highly recommend it.” Layla appreciated that she had realized that it was possible for her to be at peace even in prison and wanted to encourage as many inmates as possible to do the course—suggesting even using more extrinsic inducements if necessary.

Facilitators also commented on behavioral changes that they observed in some participants. Facilitator Emma reflected on the comments of a participant (Dana) who had expressed at the final session: “I just found out that if I’m peaceful, then other people around me are peaceful.” Emma described how Dana’s behavioral change had also been noticed by the prison program coordinator, who had acknowledged that the staff were “glad that you feel peaceful.” From Emma’s words: “I think she had been very disruptive and probably a bit, you know, difficult, and now she was on a positive track.” She added: “I think it’s had quite a remarkable effect on some of them, they’ve calmed down, and then they get . . . privileges, . . . they get rewarded for cooperative behaviour I guess.”

Bill provided the anecdotal story of a former participant who had expressed to him “my peace was taken from me many years ago, I had no idea it was still there inside of me, I found that notion so empowering.” She had previously described to him that she had felt desperate before starting the program, that she had been “at the end of the road, this course is either going to work for me, or that’s it.” Bill recollected statements that other participants had made regarding changes in their behavior (which again staff members had corroborated): “that the women are more settled, happier, and less involved in troublesome incidents within the prison. One woman said, “look, it’s settled me down so much I no longer get into trouble.” He reported that feedback from inmates and staff regarding behavioral change was positive, and that even women who could only attend one or two sessions appeared to have gained something.

Overall, there was not a considerable number of criticisms or suggestions for improvement of the program. Opinions were divided regarding length between those who thought it too long and sometimes boring (especially those who acknowledged short attention-spans), and those who thought it too short. Opinions were also divided between those who wanted more traditional educational content/materials, such as Naomi suggesting having to write “What is one thing you learnt from today,” which she thought would be a tool to minimize disengagement, a similar comment echoed by Faye. Olivia wanted homework. However, most expressed appreciation for minimal demands, such as Clair: “They let you breathe, they let you think, there’s no ulterior motive for that course.” Participants are provided workbooks where some took notes, and a coloring-in picture relevant to the topic (an adaptation created by facilitators at AWP), which some participants seemed to appreciate. The differences in learning styles of participants were thus very evident from their responses, reinforcing the need for flexibility in program facilitation.

## Discussion

The results of the quantitative and qualitative analysis reveal that participants gained better insight into inner peace, and that it led to greater self-regulation and better anger-management skills. Based on the incarcerated women’s responses, the program influenced their responses to intra-personal conflict. Many participants discussed how experiencing inner peace could improve their well-being, enable them to make better life-choices, and facilitate positive behavioral change. Some stated that they felt more peaceful and calmer after a session of the program. Some also related experiencing inner peace to acquiring greater forgiveness, both of themselves and others, and being more at peace with what they had done/being in jail. Their descriptions of an enhanced sense of self-identity enabled the imagining of positive interpersonal interactions even in stressful situations. Program facilitators confirmed the behavioral changes and progress that women reported.

Despite the paucity of literature about programs promoting inner peace, the results do however reflect some of the aspects identified in the literature review above. Whilst lacking an overt focus on conflict resolution skills (as advocated by Johnson & Johnson, 2011, 2014), several participants gave examples of how the program had facilitated greater affect-arousal control in situations of potential interpersonal conflict. This was associated with experiences of greater self-esteem and reductions in impulsivity that participants described. Reductions in affect arousal and impulsivity in tandem with cognitive changes and increases in self-esteem are potentially related to behavioral changes which could reduce offending (Fortune et al., 2014; Marshall & O’Brien, 2013). The instrumental function of anger with low self-esteem was noted by Novaco (2013), suggesting that an internal transition needs to occur from the instrumental use of violence to protect and promote self-esteem, to sourcing an alternative means of obtaining respect (presumably of oneself as well as from others).

The Risk Needs Responsivity (RNR) model describes eight core or “central” risk/need factors including impulsivity, and is based on the “General Personality and Cognitive Social Learning Perspective” (GPCSL) (Bonta & Stephen Wormith, 2013). Andrews and Bonta (2010) describe impulsivity as part of the “constellation of antisocial personality factors” (p. 6)—a major criminogenic need which is dynamic in that it is amenable to treatment. Managing reactive aggression and impulsivity can thus have a significant role in reducing the risk of reoffending. Our research indicates that the training to recognize and aspire to inner peace gave participants a clearer awareness of when they were moving to an unregulated state of reactive aggression, and created an enhanced desire and ability to self-regulate and avoid the escalation of conflict.

Fortune et al. (2014), and Marshall and O’Brien (2013) described emotional dysregulation as relating to the absence of inner peace, with the latter describing maladaptive coping responses as a significant criminogenic factor. The participants who described reduced affect arousal, which they attributed to attending the program, would appear to suggest that the acquisition of inner peace and inner resources could have some correlation with desistance, especially where impulsivity and aggression are factors. The statement of the WHO (1998) that inner peace is a “highly desirable condition” in helping people cope when things go wrong seems of particular relevance in assisting those released from prison to navigate the realities of reintegration, equipped often with minimal resources and supports.

### Limitations

This study is based on a small sample size. A small sample size is generally regarded as a limitation in seeking to achieve statistical significance, and as limiting generalisability (Maxfield & Babbie, 2017), although it can still potentially provide useful exploratory qualitative data. The data collected sheds light on how participants at AWP responded to the Peace Education Program both from their words in interview and, to a lesser extent, in their responses to the surveys. However, this data is sufficient to make some careful generalized conclusions.

Another limitation was that this research was conducted at the time of severe restrictions due to the Covid-19 pandemic. Consequently, the first author who conducted the interviews was required to change the recruitment and data collection approach, as described above, with the interviews being conducted via phone rather than in person. Also, the goal of continuing to evaluate cognitive and behavioral gains by means of a 3-month follow-up proved impracticable given the realities of the high levels of transiency of the participants and the issues with data collection due to the Covid restrictions. With any program, a follow-up would assess whether therapeutic gains are maintained, which is especially problematic in environments that are not supportive of personal growth. Finally, seeking to interview those who failed to complete the program was also beyond the scope of the present study, though this could yield rich data as to how the program could be improved.

### Future Research

Whether learning about inner peace has been internalized and will thus translate into more peaceful behaviors, either whilst in prison or on returning to the community, is an important topic for further research. A longer-term follow-up would shed light on how much any treatment gains from the program “stick” 3 or 6 months after completion. However, given the high turnover of women at AWP, such an option may only prove viable with longer-term prisoners. A longitudinal study using statistical data regarding reconviction rates, if such became available, would be beneficial. Perhaps more easily captured would be a comparison of the rates of institutional disciplinary breaches. This information could serve to provide a snapshot of behaviors and a potential point of comparison with inmates who have not attended the program. Another avenue for future research, which would avoid the issues of participants being released very shortly after completing the program, would be to conduct follow-up data collection in the community. Several of the facilitators expressed the hope that this could happen, and were perhaps disappointed that this option was beyond the scope of the present study.

## Conclusion

This study explored whether participants in the Peace Education Program gained appreciation and understanding of inner peace, and whether acquiring a greater awareness of intra-personal peace was already demonstrating any influence on inter-personal peace in practice. The participants reported that, despite the relative brevity of the program, the growth in their understanding and experience of intra-personal peace resulted in significant behavioral changes. They described greater emotional self-regulation, particularly relating to anger and its expression in interpersonal conflict. This was validated by the reports of facilitators. Participants also reported learning greater detachment and reductions in impulsivity, acquiring a more positive sense of self and becoming less reliant on the opinions of others. This further reduced their reactive aggression. Acquiring a stronger positive self-identity could also serve to reduce vulnerability to the potentially criminogenic influence of others. The program thus appears to have provided the women with an empowering alternative to a stigmatized criminal identity and pessimistic feelings of powerlessness in their challenging circumstances.

Based on the results, further research and further application of the program’s potential both to increase personal wellbeing and reduce re-offending are justified. Future iterations of the program could incorporate suggestions for improvement highlighted by participants and facilitators, particularly for ongoing renewal of the material and for a clearer means of follow-up. Further exploration could provide insights into who is most likely to benefit from the program, at what stage of their sentence, and to further explore the capacity of the program to motivate and prepare participants for the suite of official programs.

## Notes

1. Prem Rawat has received many awards for his work and has been named Ambassador of Peace four times: by the International University of Peace in Brazil; by the governor of the Basilicata region of Italy; by the National Agrarian University, Peru, and by Gianni Pittela MEP and First Vice-President of the European Parliament on behalf of the Brussels Declaration Pledge to Peace, signed at the European Parliament in 2011 (and ratified at the Italian Senate in 2012) (TPRF, 2022).
